# Impact of sports on temporomandibular dysfunction: a comparison of competitive and recreational female athletes as well as female non-athletes

**DOI:** 10.1007/s00784-022-04499-6

**Published:** 2022-04-29

**Authors:** Hannah Charlotte Freiwald, Nico Peter Schwarzbach, Anne Wolowski

**Affiliations:** Poliklinik für Prothetische Zahnmedizin und Biomaterialien, Westfälische Wilhelms-Universität Münster, Albert-Schweitzer-Campus 1/W30, 48149 Munster, Germany

**Keywords:** Temporomandibular dysfunction, TMD prevalence, Competitive sports, Sports

## Abstract

**Objectives:**

The present study was conducted to investigate the correlation between (competitive) sports and the occurrence of temporomandibular dysfunctions (TMD) by comparing the prevalences in competitive, recreational, and non-athletic women.

**Materials and methods:**

A total of 138 women between the ages of 18 and 45 were interviewed about symptoms of TMD by means of a questionnaire. Based on their athletic performance level, the participating women were classified as competitive athletes, recreational athletes, or non-athletes (each group *n* = 46).

**Results:**

Symptoms of TMD were notably less frequent in competitive female athletes (52.2%) than in recreational female athletes (63.0%) and female non-athletes (60.9%). With increasing training load, the prevalence of TMD decreased in both the competitive and recreational female athlete groups.

**Conclusions:**

Athletic activity in general seems to have a positive effect on the occurrence of TMD. Competitive female athletes appear less likely to suffer from symptoms of TMD than recreational athletes and non-athletes. One possible explanation for this could be the better supervision by qualified trainers and physiotherapists in competitive sports.

**Clinical relevance:**

Patients should be motivated to engage in sports as a protective measure against symptoms of TMD. However, it is important to ensure that they are properly instructed by experienced personnel in order to avoid unphysiological strain and negative consequences.

## Objectives

The term temporomandibular dysfunctions (TMD) refers to specific dysfunctions which affect the masticatory muscles, temporomandibular joints, and other associated structures [1]. The symptoms manifest diversely, but can typically include pain in the masticatory muscles or the temporomandibular joint, crepitations in the joint or dislocations of the articular disk, and restricted or asymmetric mandibular movements [1–3].

The German Society of Craniomandibular Function and Disorders recommends that TMD patients engage in sports, especially endurance sports, as a form of complementary self-therapy [4]. An extensive literature review was already conducted to scientifically investigate whether competitive athletes are protected from TMD by their intense athletic activity or whether the double burden of pursuing an athletic career in addition to their education or profession might even have a TMD-promoting effect [5]. Although only limited research literature is currently available on this topic, the existing data suggests that competitive athletes suffer from TMD more frequently [5]. In competitive sports, techniques are constantly being improved to further increase peak performance. In order to examine the relationship between TMD and competitive sports in more detail, and thus to be able to detect and prevent possible negative effects on the athletes’ orofacial area and their performance at an early stage, the present study was conducted to compare TMD prevalences in competitive female athletes, recreational female athletes, and non-athletic women.

## Materials and methods

### Type of survey

In order to recruit female subjects, requests were sent to sports clubs, gyms, universities, and similar institutions throughout Germany between November 2019 and October 2020. Attempts were made to contact well-known German sports clubs. All clubs that gave feedback were contacted. If they consented to participate in the survey, the facilities were visited in person by the author. After presenting a brief overview of the study, she handed out written patient information to the potential participants. The questionnaire was then issued to those who agreed to take part in the study. While filling out the form, the subjects were given the opportunity to ask the examiner for clarification at all times. Based on this strategy, female athletes from all over Germany could be included in the survey. A total of 188 questionnaires were issued. Since this is a pilot study, various sports and a broad age spectrum were included. The aim of the study was to give an overview of the present topic.

### Inclusion criteria

All women who were of legal age but no older than 45 years at the time of the survey were eligible to participate in the study.

### Exclusion criteria

Minors and pregnant women were not allowed to participate in the survey as well as those older than 45 on the day of the survey. People with mental disabilities or people with a legal representative were also excluded. Since both the information handout and the questionnaire itself were in German, people who were not able to speak and write German were excluded.

### Questionnaire

To ensure that only female subjects between the ages of 18 and 45 participated in the study, the first questions asked were about gender and age.

In order to categorize the subjects according to their athletic performance level, the subsequent questions assessed their type of sport and training schedule. All subjects who exercised more than twice a week were considered athletes, while participants who exercised less or not at all were categorized as non-athletes. To further differentiate the athletes, their training frequency was inquired, but only workouts of at least 1 h were counted. Competitive and recreational female athletes were categorized based on the following question: “Have you competed in at least statewide athletic competitions in the past 6 months?” Participants who answered “yes” were categorized as competitive athletes. In order to examine the training behavior in more detail, the athletes were asked questions about the extent of their strength and endurance training. The last question on the athletic aspect was focused on the athletic performance during the past 6 months.

In order to identify possible signs of temporomandibular dysfunction, the following aspects were addressed:

- Pain in the jaw or temporomandibular joints,
- Audible or perceptible clicking or crepitus in the temporomandibular joints during mouth movements
- Limitations and/or discomfort associated with chewing, opening, closing, and/or lateral jaw movements
- Asymmetry of mouth opening
- Maximum mouth opening
- Muscle hardening and/or muscular pain around the temple, cheek, and/or jaw angle.

Those who answered “yes” to at least one of the above questions were subcategorized into a group of participants who had symptoms that possibly indicated a TMD.

### Data analysis

The results of the survey were entered into an Excel spreadsheet and analyzed using the statistical software R (R version 4.0.2). The chi-squared test for independence was used to statistically calculate the correlations between athletic behavior and the occurrence of TMD symptoms. The significance level was set to *α* = 5%. The assumed null hypothesis was there is no correlation between competitive sports, recreational sports, or physical inactivity and the prevalence of symptoms associated with TMD.

### Application for ethical approval

The conducted study complies entirely with ethical principles. It was approved by the Ethics Committee of Westfalen-Lippe in Germany (2019–224-f-S). Participants were informed that they could withdraw their consent to the study at any time without stating a reason. Opting out would not have any consequences.

## Results

Of the 188 questionnaires distributed, two could not be included in the evaluation because the required minimum age was not met or the information was not sufficient for a valid analysis. The aim of the planned study was to draw a comparison between competitive and non-competitive female athletes. Therefore, a statistically sample size planning was accomplished with the program ADDPLAN, Version 6.0.9. The planning revealed a number of 93 participants per group. This article examined competitively athletic, recreationally athletic, and non-athletic women. The number of cases was adjusted to the smallest subgroup, i.e., recreationally athletic women. To this end, 46 competitively athletic and 46 non-athletic women were selected randomly, while all 46 recreationally athletic women were included.

Except for a few isolated questions, all of the questionnaires were filled out completely. Since it was still possible to clearly assign the test subjects, they could be included in the evaluation.

The competitive athletes engaged in the following sports: handball, soccer, volleyball, lifeguarding, open water swimming, swimming, triathlon, judo, Latin dance, and rowing. All these athletes were trained by professional coaches and received physiotherapeutic support. Among the recreational athletes, gym training predominated, but the following sports were listed as well: basketball, handball, lacrosse, soccer, tennis, swimming, fencing, garde dancing, golf, judo, salsa, aerobics, crossfit, cycling, jogging, step aerobics, weight training, and yoga.

The average age in the group of female competitive athletes was 23.8 years. The recreational athletes and non-athletes had an average age of 25.5 and 27.7 years, respectively. The majority of the participants had graduated from high school (German “Abitur”), so that all three groups had a comparable level of education.

### Frequency of TMD symptoms

In the group of competitive athletes, symptoms indicating TMD were present in 52.2% of the women (24 out of 46 subjects). This value was lower than it was in the group of recreational athletes (63.0%; 29 out of 46 women). At 60.9% (28 out of 46 subjects), the control group of non-athletes ranged in between.

The chi-square test of independence yielded an *χ*^2^-value of 1.2554 at a significance level of 5%. The corresponding critical value of the chi-square distribution table is 5.99. As this is higher than the calculated *χ*^2^-value, the null hypothesis cannot be rejected (*p*-value = 0.5338).

### Distribution of TMD symptoms

The data on the relative distribution of TMD symptoms refers to both the subgroup of those who reported TMD symptoms as well as to the respective complete group (*n* = 46) of competitive, recreational, or non-athletes.

#### Group of competitive athletes

At 70.8% and 37.0%, respectively, clicking or grating sounds in the temporomandibular joint (TMJ) were among the most common TMD symptoms among female competitive athletes. Soreness or hardening of the masticatory muscles (45.8% and 23.9%, respectively) and limited or painful jaw opening (41.7% and 21.7%, respectively) were also reported frequently.

#### Group of recreational athletes

Among the female recreational athletes, again, clicking and grating sounds in the temporomandibular joint were the predominant symptoms (58.6% and 37.0%, respectively), followed by pain around the jaw or temporomandibular joints (55.2% and 34.8%, respectively). The third most frequent TMD symptom was an asymmetric jaw opening, which was found in 51.7% and 32.6%, respectively, of the female recreational athletes.

#### Group of non-athletes

In the group of non-athletes, clicking or grating noises in the TMJ were the most common TMD symptoms as well (67.9% and 41.3%, respectively). Pain around the jaw or temporomandibular joints occurred similarly often (64.3% and 39.1%, respectively).

**Diagram 1:**
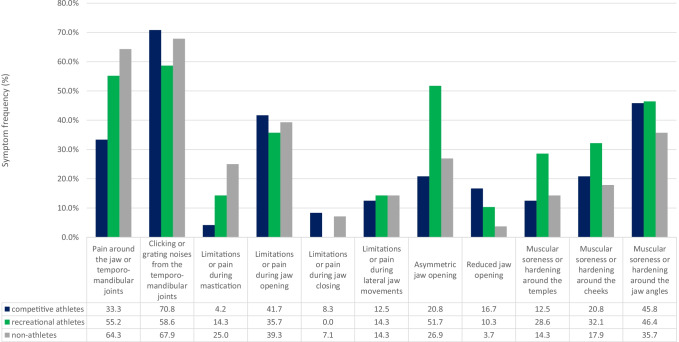
Distribution of TMD symptoms with respect to the subgroups of subjects with TMD symptoms

**Diagram 2:**
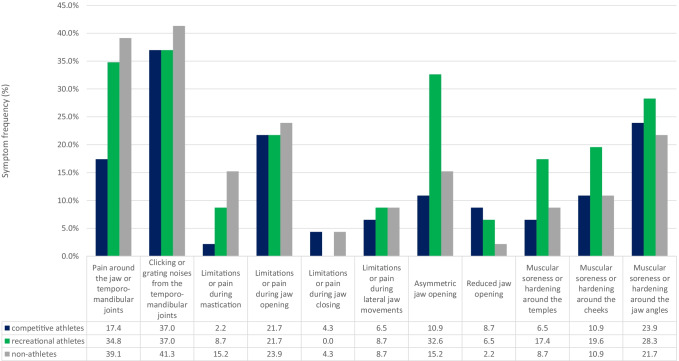
Distribution of TMD symptoms with respect to the total groups

The frequency of all TMD symptoms are shown in Diagrams 1 and 2.

### The impact of training

#### Effects of training frequency

Of the competitive athletes who routinely completed three to five workouts of at least 1 h each on a weekly schedule, 61.5% showed symptoms of TMD. Of those training at least six times per week, 16 (48.5%) out of 33 women reported TMD symptoms.

While 70.0% of the recreational athletes who exercised three to five times a week for at least 1 h each reported TMD symptoms, three of the four subjects (75.0%) who completed at least six workouts a week had no symptoms.

All figures pertaining to training frequency are presented in Table 1.

**Table 1:** Distribution of competitive and recreational female athletes on the occurrence of TMD symptoms in relation to the weekly amount of training, on the occurrence of TMD symptoms in relation to the weekly amount of weight training, on the occurrence of TMD symptoms in relation to the weekly amount of endurance training, and on the occurrence of TMD symptoms in relation to their athletic performance during the preceding 6 months

	Competitive athletes				Recreational athletes			
	TMD symptoms (*n* = 24)		No TMD symptoms (*n* = 22)		TMD symptoms (*n* = 29)		No TMD symptoms (*n* = 17)	
	Relative percentage	Absolute number	Relative percentage	Absolute number	Relative percentage	Absolute number	Relative percentage	Absolute number
Number of workouts per week								
3–5	61.5	8	38.5	5	70.0	28	30.0	12
6 or more	48.5	16	51.5	17	25.0	1	75.0	3
Number of weight training sessions per week								
0	50.0	1	50.0	1	66.7	4	33.3	2
1–2	51.4	18	48.6	17	63.6	14	36.4	8
3–5	50.0	4	50.0	4	57.1	8	42.9	6
6 or more	0.0	0	0.0	0	66.7	2	33.3	1
Number of endurance workouts per week								
0	100.0	1	0.0	0	83.3	5	16.7	1
1–2	41.2	7	58.8	10	50.0	12	50.0	12
3–5	73.3	11	26.7	4	76.9	10	23.1	3
6 or more	33.3	4	66.7	8	0.0	0	100.0	1
Athletic performance during the preceding 6 months								
Above average	53.3	8	46.7	7	66.7	4	33.3	2
Average	46.4	13	53.6	15	56.0	14	44.0	11
Below average	100.0	2	0.0	0	78.6	11	21.4	3

#### Effects of weight training

The competitive athletes showed a similar distribution for zero, one to two, and three to five weekly weight training sessions: approximately 50% showed TMD symptoms.

The recreational athletes who did not engage in weight training had TMD symptoms in 66.7%. Of those who completed one to two weight training sessions per week, 63.6% reported symptoms indicating TMD. Those who did weight training three to five times a week showed TMD symptoms with a frequency of 57.1%. Only three female recreational athletes performed weight training at least six times per week; two of them reported TMD symptoms.

For an overview of all figures pertaining to weight training, refer to Table 1.

#### Effects of endurance training

Only one competitive athlete did not perform any routine endurance training; she did show TMD symptoms. Seventeen competitive athletes pursued endurance training once or twice a week. Of these subjects, 41.2% reported TMD symptoms. Of those who completed three to five endurance training units per week, 73.3% also experienced TMD symptoms. Out of the 12 female competitive athletes who completed endurance-focused training units at least six times per week, 33.3% suffered from TMD symptoms.

Five of the six female recreational athletes (83.3%) who did not do endurance training reported TMD symptoms. At one to two weekly endurance units, half of the recreational athletes were experiencing TMD symptoms. Among those who performed endurance training three to five times a week, TMD symptoms occurred with a frequency of 76.9%.

All data on endurance training is shown in Table 1.

#### Effects on athletic performance

Taking into account their athletic performance of the previous 6 months, 53.3% of the female competitive athletes with above-average performance showed TMD symptoms. Of those who had recently performed at an average level, 46.4% reported symptoms indicating TMD. Only two female competitive athletes rated their athletic performance as below average. Both of them experienced TMD symptoms.

Among the recreational athletes who had recently performed above average, TMD symptoms occurred with a frequency of 66.7%. Of those performing at an average athletic level, 56.0% reported symptoms of TMD. Those women who rated their athletic performance as below average in the last 6 months showed TMD symptoms in 78.6%.

Table 1 shows all the figures pertaining to athletic performance.

## Discussion

### Frequency of TMD symptoms

The obtained results indicate that competitive female athletes are considerably less likely to be affected by TMD symptoms than recreational female athletes (52.2% compared to 63.0%). Remarkably, the non-athletes, 60.9% of whom reported symptoms of TMD, ranked close to the recreational athletes. Comparable values for competitive athletes were found in the study by Bonotto et al. [6], which included both female and male subjects. In that study, 54.2% of the competitive karateka showed temporomandibular dysfunction [6]. Somewhat higher TMD frequencies in competitive athletes were identified in studies by Mendoza-Puente et al. [7] (boxers: 77.77% and boxers and handball players combined: 60.53% at least moderate TMD) and Bonotto et al. [6] (competitive mixed martial arts athletes: 61.5%). For competitive handball players, Mendoza-Puente et al. [7] detected a slightly lower TMD score of 45.00%. As determined by Weiler et al. [8] (basketball players: 26%) and Weiler et al. [9] (female basketball and handball players: 16.85%), temporomandibular dysfunctions were considerably less frequent in competitive athletes. The recreational athletes in the present study scored notably higher TMD frequencies compared to those found by Bonotto et al. [6] for amateur karateka (17.6%), by Zamora-Olave et al. [10] for male and female field hockey players (11.7%), and by Zamora-Olave et al. [11] for male and female water polo players (20.2%). This is also true for the frequency of TMD symptoms in non-athletic women: Weiler et al. [8] (12%), Weiler et al. [9] (11.11%), and Bonotto et al. [6] (14.3%) reported notably lower values here as well. Study results on the average population regardless of the subjects’ athletic performance contain values similar to the frequencies determined here, as well as considerably lower values. Namely, in the third German oral health study [12], TMD symptoms were detected anamnestically in 21.3% of the females and males combined and in 26.0% of the females evaluated separately, whereas clinically, 51.1% of all subjects and 55.8% of the females showed TMD. Heß [13] determined temporomandibular dysfunctions in 21.5% of the female subjects. The study by Barbosa et al. [14] yielded TMD frequencies of 39.3% of all subjects and 41.7% of the females.

As Freiwald et al. [5] demonstrated, the comparability of the different sports-related studies [6–11] is limited. On the one hand, the research methods and case numbers of the studies differ greatly in some cases, and on the other hand, a wide variety of sports were examined [5]. In the current study, anamnestic data was used to screen for TMD symptoms. No confirmed diagnoses were established, but as a guideline, a general overview of present symptoms which were possibly indicating a manifest TMD was given. This could be a factor contributing to the higher TMD prevalence compared to other studies. Moreover, in the studies, men and women are evaluated both separately [7–9] and together [6, 10, 11]. As has been demonstrated in various studies, TMD prevalence is higher in women than in men [2, 12–20]. For this reason, this study included only women. This also provides a rationale for the higher TMD frequencies compared to some other studies. Age also plays an important role in the prevalence of temporomandibular dysfunction, as supported by a large number of studies [12, 14, 17–19]: young adults are affected most severely, whereas younger and older persons are less likely to suffer from TMD symptoms. The present study included women aged between 18 and 45 years (mean age: 23.8 (competitive athletes), 25.5 (recreational athletes), and 27.7 (non-athletes) years), the age group most commonly affected by TMD. From that, another explanation for the high TMD values can be discerned. According to Barbosa et al. [14], persons older than 25 years have a higher risk of developing TMD than younger persons, which, in addition to the athletic aspect, may be another reason for the higher TMD prevalence among recreationally athletic and non-athletic women.

In contrast to the previously available literature, which was compiled and evaluated in an article by Freiwald et al. [5], the present study found that competitive female athletes are less frequently affected by TMD symptoms. Statistically, no significant correlation between the level of athletic performance and the occurrence of TMD symptoms could be established; however, the relative frequencies do reveal a tendency. Competitive sports seem to have a protective effect regarding the development of TMD symptoms. Recreational female athletes indicated TMD symptoms considerably more often than competitive female athletes and slightly more often than non-athletes. This suggests that female recreational athletes may be at greater risk for injury related to incorrect exercise and overstraining due to lack of supervision by a qualified team of instructors and physiotherapists, resulting in a higher incidence of TMD symptoms [21].

### Distribution of TMD symptoms

Both the most and the least frequent symptoms of temporomandibular dysfunction are similarly distributed in the groups of competitive, recreational, and non-athletic women. Strikingly, however, female recreational athletes were notably more likely to report having an asymmetrical mouth opening (recreational athletes: 51.7% and 32.6%, respectively; competitive athletes: 20.8% and 10.9%, respectively; non-athletes: 26.9% and 15.2%, respectively). Pain around the jaw or temporomandibular joints was reported by 55.2% and 34.8%, respectively, of the female recreational athletes and by as many as 64.3% and 39.1%, respectively, of the non-athletes, but by only close to half as many of the female competitive athletes (33.3% and 17.4%, respectively).

#### Pain around the jaw or temporomandibular joints

Of all competitive female athletes, 17.4% suffered from pain around the jaw or temporomandibular joints, while among the recreational female athletes, this symptom was found in 34.8% of the cases. Of the non-athletic women in this study, 39.1% were considered symptomatic in this respect. The study by Persson et al. [22] examined the frequency of tenderness around the TMJ in wrestlers and persons engaged in sports other than wrestling or no sports at all. In 7.69% of the wrestlers and none of the non-wrestlers, symptoms were found anamnestically, whereas clinically, symptoms occurred in 3.85% of both groups, respectively [22]. Thus, the figures determined in this study are considerably higher than those of Persson et al. [22]. However, no information on athletic performance level was provided in the study [22]. A study by Gay-Escoda et al. [23] delivered similarly low values; here, pain around the jaw or temporomandibular joints was found in 6.7% of professional soccer players. But again, as described above for the general prevalence of TMD symptoms, the fact that both studies included only male subjects could be a possible explanation for the low symptom frequencies. In the study by Soares et al. [24], 17% of male and female students aged 18 to 30 years exhibited pain in the temporomandibular joint. The value of this sample of the general population is thus similar to the prevalence in female competitive athletes, but considerably lower than the frequencies in recreational athletes and non-athletes. In a group of TMD patients, Manfredini et al. [3] demonstrated temporomandibular joint pain in 40.6% of the subjects. Regarding the frequency distribution within the group of those who experienced TMD symptoms, competitive athletes had somewhat lower values (33.3%), while recreational athletes (55.2%) and non-athletes (64.3%) showed increased values.

#### Clicking or grating noises from the temporomandibular joints

The prevalence of temporomandibular joint crepitus was similar in all three groups: 37.0% of both competitive and recreational female athletes reported this symptom, while among non-athletes it occurred in 41.3% of the subjects. For (competitive) athletes as well as for non-athletes, Weiler et al. [8], Weiler et al. [9], Persson et al. [22], and Gay-Escoda et al. [23] found notably lower values: basketball players 4.3%, female basketball and handball players 4.49%, wrestlers 15.38%, soccer players 16.7%, and non-athletes (male and female) 2.4 to 3.85%. Bonotto et al. [6] produced comparable to higher values for competitive athletes, 45.8% (competitive karatekas) and 38.5% (competitive MMA athletes). However, in the same investigation, the prevalences of 11.8% in recreational athletes (amateur karatekas) and 7.1% in non-athletes were considerably lower than the values determined for the corresponding groups in this study. In the average population without differentiation according to athletic performance, the third German Oral Health Study [12] indicated that crepitus in the temporomandibular joint was found through clinical examination in 39.4% of female subjects between the ages of 35 and 44 years. This value ranges between the values determined for the three groups in this study. Heß [13] showed that within a group of TMD patients, clicking noises were found in 58.2%, grating in 12.3% of the subjects. Similarly, the subgroup of female recreational athletes with TMD symptoms reported clicking or grating noises in the temporomandibular joint in 58.6%. In this respect, the corresponding groups of non-athletes (67.9%) and competitive athletes (70.8%) had higher values. Manfredini et al. [3] determined a frequency of 54%, which is similar to the prevalence found in recreational female athletes in the present study. At 48.9% among TMD patients, Osiewicz et al. [2] also reported the prevalence of this symptom to be in the same range. The frequency of temporomandibular joint crepitus in the general population is similar to the values of the recreational female athletes in this study. An explanation for this probably lies in the high percentage of recreational athletes in the population [25].

##### Asymmetric mouth opening

While competitively athletic (10.9%) and non-athletic (15.2%) females were relatively unlikely to report an asymmetrical mouth opening, recreational athletes (32.6%) were more than twice as likely to be affected. Persson et al. [22] found values comparable to competitive female athletes in wrestlers (11.54%) and comparable to non-athletes in non-wrestlers (19.23%). Studies by Weiler et al. [8] and Gay-Escoda et al. [23] reported slightly lower values for competitive athletes (basketball players (6.5%) and soccer players (6.7%)). In contrast, Weiler et al. [9] identified deviations in the mouth opening in only 1.12% of female basketball and handball players. At 2.4% in men [8] and 2.78% in women [9], the frequencies among non-athletes are also considerably lower than the ones in this study. If only the subgroups of females with TMD symptoms are considered, Weiler et al. [8] provided values for basketball players (25.0%), which are similar to those obtained for female competitive athletes (20.8%) in the present study. Weiler et al. [9] found asymmetric mouth opening in 25.00% of non-athletic women, which is close to the values of non-athletic women (26.9%) determined here. For the general population, Heß [13] reported deviations in 15.7% and deflections in 19.9% of the subjects with TMD. These values are well below those of the recreational female athletes (51.7%) and the non-athletes in this study.

#### In general

The frequency distribution of the individual symptoms supports the hypothesis previously made for the general prevalence of TMD symptoms: competitive female athletes seem to be less likely to suffer from TMD symptoms than recreational female athletes.

### The impact of training

#### Effects of training frequency

Competitive female athletes who trained three to five times a week were considerably more likely to report TMD symptoms than those who trained at least six times a week (61.5 to 48.5%). In the latter group, more than half of the female athletes were even symptom free (51.5%). Similarly, among the recreational athletes, those who exercised three to five times a week were by far more likely to experience TMD symptoms than those who exercised more often (70.0 to 25.0%). However, as only four female recreational athletes trained at least six times per week, the small number of subjects reduces the reliability of this evaluation. Most studies that analyzed TMD prevalence among athletes either did not report training frequency at all [10, 11, 22, 26] or only stated a total number of hours per week [6, 8, 9], providing limited reference values. Although Gay-Escoda et al. [23] indicated that the examined professional soccer players trained four to five days per week, they did not specify overall TMD prevalence but listed the frequencies of individual symptoms. Only the study by Mendoza-Puente et al. [7] contains such data. The boxers and handball players in that investigation exercised at least five times a week, and 60.53% of them showed at least moderate TMD [7]. This means that those competitive athletes can be compared to the female competitive athletes in this investigation, who trained three to five times a week.

Since TMD symptom frequency decreases with increasing training workload in both the competitive and recreational female athlete groups, extensive training appears to have a favorable effect on the occurrence of TMD symptoms. Out of all subjects who exercised three to five times a week, the competitive athletes were less likely to be affected by TMD symptoms than the recreational athletes. One reason for this could be the more thorough supervision that female competitive athletes receive from their trainers and physiotherapists. Thanks to the stricter monitoring of correct training execution, female competitive athletes seem to be more effectively protected from overload or unphysiological strain than female recreational athletes, who practice their sport independently and without receiving corrections from experienced trainers [21].

#### Effects of weight training

Whether the female competitive athletes performed no weight training, one to two, or three to five weight training sessions per week, the probability of TMD symptoms occurring was about 50% in each case. Thus, the amount of weight training does not seem to influence the occurrence of TMD symptoms in female competitive athletes. However, the situation is different for the female recreational athletes, where TMD symptom frequency steadily decreases from no weight training (66.7%) over one to two weight training sessions (63.6%) to three to five weight training sessions (57.1%) a week. Two factors could contribute to this effect. Firstly, those who perform weight training three to five times a week also exercise more in general, so the positive effect of increased training workload described above could have an effect. And secondly, it seems natural that a person who frequently performs weight training will also be more experienced in its correct execution, so that there occurs less unphysiological strain. The currently available literature provided no data on the relationship between TMD prevalence and weight training frequency.

#### Effects of endurance training

Female competitive athletes who performed endurance training once or twice a week were considerably less likely to experience TMD symptoms than those who trained three to five times a week (41.2 to 73.3%). Even more endurance training was associated with a decrease in TMD symptoms to 33.3% among the female competitive athletes. Female recreational athletes who did not engage in endurance training at all or who did so three to five times a week were notably more likely to exhibit TMD symptoms than those who trained once or twice a week (83.3% and 76.9 to 50.0%, respectively). Any endurance training that is performed makes TMD symptoms much less likely to occur. In both groups, TMD symptom frequencies were lower, when the training load was less than three to five endurance units per week. These findings suggest that endurance training has a positive effect on the occurrence of typical TMD symptoms, as long it is not excessive. The marked decrease in symptom frequency among female competitive athletes who performed at least six endurance units could be attributed to these athletes’ special adaptation to extensive training. The German Society of Craniomandibular Function and Disorders recommends that TMD patients engage in light athletic activity, especially endurance-focused sports [4]. This corresponds well with the positive effect of one to two endurance training units per week highlighted in this investigation. Similar to weight training, no data on the relationship between endurance training and TMD frequencies could be found in the currently available literature.

#### Effects on athletic performance

When looking at the athletic performance of the competitive and recreational female athletes, it became apparent that a considerable majority of those who rated their performance as below average also reported TMD symptoms (competitive female athletes 100%, recreational female athletes 78.6%). It should be noted, however, that only two female competitive athletes stated below-average performance. Such a strong correlation is not found for either above-average or average performance. On the one hand, it seems conceivable that the presence of TMD symptoms impairs athletic performance. On the other hand, TMD symptoms could also have originated from severe psychological stress outside the athletic environment and subsequently developed into an additional factor that negatively affected athletic performance. Studies by Kanehira et al. [27], Kmeid et al. [20], and Wieckiewicz et al. [28] have already demonstrated the associations between psychoemotional burdens or stress and TMD.

### Limitations

The present study is a pilot study. Currently, there is still very little literature available on the topic of TMD and sports, as was already pointed out in the literature review of Freiwald et al. [5]. In order to obtain an overview of the relationship between TMD and the subjects’ sports habits, this study included different types of sports. On the one hand, this resulted in a heterogeneous group of subjects, but on the other hand, it provided some preliminary hints about the relationship between TMD and (competitive) sports. Since none of the included sports directly affects the facial and maxillary region, direct effects of sports equipment such as diving mouthpieces could be ruled out. Furthermore, when interpreting the results, it should also be taken into account that the data was collected using a questionnaire. Even though the subjects were shown how to assess aspects of the questionnaire pertaining to their own body and had the opportunity to ask questions during the whole process, clinical examinations by trained and experienced staff do provide more accurate results. However, because the present study is the first of its kind, a conscious decision was made for a questionnaire similar to the TMD screening tests instead of clinical examinations. In order to investigate the effects of the different sports in more detail, follow-up studies are advised. Subsequently, clinical examinations are recommended to validate these effects. This will allow for more precise statements, which, due to its design, cannot yet be drawn from this pilot study. The results of this present study are thus to be seen as trend-setting for further investigations on the topic.

## Conclusions

As this study was designed as a pilot study, the results should be seen as an approximation of the relationship between TMD and the subjects’ sports habits.

Contrary to what the available literature suggested, the present study found that competitive female athletes appear to be less frequently affected by TMD symptoms than recreational female athletes. One possible explanation could be that female competitive athletes receive better supervision from trainers and physiotherapists, especially in terms of proper instructions for the various training exercises, well-structured training plans for the long term, and the ability to act quickly when overstrain is imminent. In contrast to recreational sports, where athletes train independently and for the most part without supervision by experienced coaches, unphysiological strain and its potential impact on the orofacial region and temporomandibular dysfunction can thus be avoided in the field of competitive sports.

As literature on sports and TMD prevalence remains scarce, it is worth investigating the subject with further studies. One approach to this would be to elaborate on the influence of a qualified trainer, for example, by comparing TMD frequencies in recreational athletes before and after receiving instructions.

## Clinical relevance

As athletic activity seems to have a favorable effect by protecting against the development of TMD symptoms, it should be recommended to TMD patients. However, it is important to ensure that patients are instructed in their respective sports by experienced personnel in order to avoid unphysiological strain and negative consequences.

## Data Availability

All data generated or analyzed during this study is included in this published article and its supplementary information files.

## Abbreviations

et al.: And others
i.e.: Id est (that is)
MMA: Mixed martial arts
*n*: Case number
TMD: Temporomandibular dysfunctions
TMJ: Temporomandibular joint
